# Preparation and Characterization of Bio-Based PLA/PBAT and Cinnamon Essential Oil Polymer Fibers and Life-Cycle Assessment from Hydrolytic Degradation

**DOI:** 10.3390/polym12010038

**Published:** 2019-12-25

**Authors:** Zormy Nacary Correa-Pacheco, Jaime Daniel Black-Solís, Pedro Ortega-Gudiño, Marcos Antonio Sabino-Gutiérrez, José Jesús Benítez-Jiménez, Alfonso Barajas-Cervantes, Silvia Bautista-Baños, Liliana Beyalith Hurtado-Colmenares

**Affiliations:** 1 CONACYT-Centro de Desarrollo de Productos Bióticos. Instituto Politécnico Nacional, Carretera Yautepec-Jojutla, km 6, calle CEPROBI, No. 8, San Isidro, Yautepec, Morelos 62731, Mexico; 2 Centro de Desarrollo de Productos Bióticos. Instituto Politécnico Nacional, Carretera Yautepec-Jojutla, Km. 6, calle CEPROBI No. 8, San Isidro, Yautepec, Morelos 62731, Mexico; blackjdec1@hotmail.com (J.D.B.-S.); silviabb2008@hotmail.com (S.B.-B.); 3 Departamento de Ingeniería Química, Centro Universitario de Ciencias Exactas e Ingenierías, Universidad de Guadalajara, Blvd. Gral. Marcelino García Barragán # 1451, Guadalajara, Jalisco 44430, Mexico; pedroo.cucei@gmail.com (P.O.-G.); alfonso.bcervantes@alumnos.udg.mx (A.B.-C.); 4 Departamento de Química, Grupo B5IDA, Universidad Simón Bolívar, Apartado 89000, Caracas C. P. 1080-A, Venezuela; msabinog@gmail.com (M.A.S.-G.); lilianahurtado10@gmail.com (L.B.H.-C.); 5 Instituto de Ciencia de Materiales de Sevilla, CSIC-Universidad de Sevilla, Avda. Américo Vespucio 49, Isla de la Cartuja, 41092 Sevilla, Spain; benitez@icmse.csic.es

**Keywords:** bio-based fibers, cinnamon essential oil, differential scanning calorimetry, mechanical properties, hydrolytic degradation

## Abstract

Nowadays, the need to reduce the dependence on fuel products and to achieve a sustainable development is of special importance due to environmental concerns. Therefore, new alternatives must be sought. In this work, extruded fibers from poly (lactic acid) (PLA) and poly (butylene adipate-co-terephthalate) (PBAT) added with cinnamon essential oil (CEO) were prepared and characterized, and the hydrolytic degradation was assessed. A two-phase system was observed with spherical particles of PBAT embedded in the PLA matrix. The thermal analysis showed partial miscibility between PLA and PBAT. Mechanically, Young’s modulus decreased and the elongation at break increased with the incorporation of PBAT and CEO into the blends. The variation in weight loss for the fibers was below 5% during the period of hydrolytic degradation studied with the most important changes at 37 °C and pH 8.50. From microscopy, the formation of cracks in the fiber surface was evidenced, especially for PLA fibers in alkaline medium at 37 °C. This study shows the importance of the variables that influence the performance of polyester-cinnamon essential oil-based fibers in agro-industrial applications for horticultural product preservation.

## 1. Introduction

In the last few decades, there has been a growing interest in the use of biodegradable materials for food packaging instead of conventional petroleum-based plastics [1]. Among all sustainable polymers, poly (lactic acid) (PLA) is one of the most promising [2]. It is a biocompatible, biodegradable, and FDA (Food and Drug Administration)-approved and generally recognized as safe (GRAS) for use in contact with food, making it a good alternative as an environmentally friendly packaging material to extend the shelf life of fruit and vegetables [3]. However, PLA has the disadvantage of being brittle [4]. Thus, it has been blended with other polymers of high elasticity such as poly (butylene adipate-co-terephthalate) (PBAT). PBAT is considered a sustainable green polyester, of which its compostability offers a sustainable disposal alternative [5]. PLA/PBAT blends have demonstrated excellent physico-chemical and mechanical properties, being employed for different applications in medicine, industry, and packaging [6,7,8,9,10,11].

Moreover, the incorporation of bioactive agents with known antimicrobial activity like essential oils into PLA/PBAT blends are proven to extend the shelf life of agricultural products [12,13,14]. Few studies have been reported in the literature related to the use of essential oils incorporated into PLA, PBAT, or their blends [15,16,17,18]. Marcos et al. [19] developed biodegradable films with antioxidant properties based on Ecoflex^®^ (BASF’s trade name for PBAT), PLA, α-tocopherol, and olive leaf extract for packaging applications. Antioxidant capacity, color, mechanical properties, and in vitro assays were performed. Cardoso et al. [20] developed active PBAT films incorporating oregano essential oil for fish fillet preservation. The obtained films were suitable for food packaging applications due to their good properties found and to control microbial growth. On the other hand, PLA-based composite films containing rosemary, myrtle, and thyme essential oils were prepared by solvent casting [21]. Mechanical properties improved with the addition of the essential oil, and the addition of 1.5% commercial thyme and 5% natural myrtle oils, increased the antifungal activity against *Aspergillus niger*.

Antimicrobial activity of cinnamon essential oil (CEO) for food preservation has been tested against many fungi and bacteria as a natural an economic alternative to conventional fungicides and bactericides. The physicochemical, barrier, mechanical, and antimicrobial activity of sago-starch films incorporated with cinnamon essential oil and TiO_2_ nanoparticles were studied by Arezoo et al. [22]. They found that the mechanical properties were improved and the bionanocomposite showed an important antibacterial activity against *Escherichia coli*, *Salmonella Typhimurium*, and *Staphylococcus aureus*. They suggest a potential application of these films for active packaging of fresh pistachio. Zhang et al. [23] characterized a multi-layer antibacterial film elaborated from chitosan and sodium alginate and added with cinnamon essential oil. The multilayer film exhibited higher retention and release of CEO compared to a single layer film and a noticeable inhibition of expansion of *Penicillium* on apple fruit after 15 days of inoculation. Although many studies have been conducted about blending and mechanical properties of PLA/PBAT films, application of PLA/PBAT with antimicrobials like CEO in food packaging is a new research subject that should be explored. In a previous work, our research group studied the antifungal activity of PLA/PBAT non-woven nets with CEO for the control of *Alternaria alternata* in tomatoes with the objective of improving their shelf life. From the in vitro results, it was found that fibers with 6.1% CEO inhibited the mycelial growth of *A. alternata* by 72.7% and their germination by 100% [24].

On the other hand, PLA can be degraded under soil conditions, hydrolysis, oxidative, and trans-esterification reactions and under processing conditions [25,26]. The main hydrolytic reaction for PLA takes place in the amorphous zones of the polymer. This is followed by chain scission with carboxyl groups affecting the reaction catalytically [27]. In aqueous solution, the hydrolytic mechanism proceeds through cleavage of the ester bond. This is controlled by the rate constant, absorbed water, diffusion coefficient of chain fragments, and solubility of degradation products [4]. Many authors have studied hydrolytic degradation of PLA [28,29,30]. However, few studies have been conducted to study hydrolytic degradation of PBAT and PLA/PBAT blends with CEO. Therefore, the life-cycle assessment of such degradation is of special interest.

The main application proposed for the present fibers is as nets for packaging agricultural commodities to preserve their shelf life in environments subjected to changes in temperature and high humidity during transportation and storage in supermarkets. That is why the characterization and study of the main factors affecting the hydrolytic degradation is important. In this research, PLA as biodegradable polymer and PBAT as flexible compostable polymer were blended in different ratios and CEO was incorporated as plasticizer and antimicrobial agent. Therefore, the objective of this work was to prepare and characterize bio-based PLA/PBAT and CEO fibers through scanning electron microscopy (SEM), Fourier transform infrared spectroscopy (FTIR), differential scanning calorimetry (DSC), and the measurement of the mechanical properties. Moreover, the hydrolytic degradation at different pH and temperatures, changes in viscosity, and morphology during the process were assessed.

The contamination of fruit and vegetables with pathogenic microorganisms causes important annual losses in the food industry. In addition, there are new regulations regarding the use of non-biodegradable materials in the conservation of food from agriculture; thus, the concept of using a packaging made from biodegradable polymer fibers added with antimicrobial bioactive agents such as essential oils, will avoid the use of synthetic fungicides that pollute the environment. Therefore, the final product application as nonwoven nets from the fibers, represents a lightweight, visually attractive packaging option, with an antimicrobial and product protection function, in addition to avoiding environmental contamination.

## 2. Materials and Methods

### 2.1. Materials

Poly (lactic acid) (PLA, 7001D) with a specific gravity of 1.24 g cm^−3^ and a melt flow rate (MFR) of 6 g 10 min^−1^ (210 °C, 2.16 kg) and poly (butylene adipate-co-terephthalate) (PBAT, Ecoflex^®^ F Blend C1200) with a specific gravity of 1.25–1.27 g cm^−3^ and a melt flow rate (MFR) of 2.7–4.9 g 10 min^−1^ (190 °C, 2.16 kg) were bought from NatureWorks (Minnetonka, MN, USA) and BASF (Mexico City, Mexico), respectively. Cinnamon bark essential oil (*Cinnamomum zeylanicum*) (CEO) was purchased from dōTERRA (Mexico City, Mexico) and glycerol (G) was bought from J.T. Baker (Mexico City, Mexico). The degree of purity for CEO and glycerol was 99.8% and 100%, respectively.

### 2.2. Blend and Fiber Preparation

PLA and PBAT were dried at 60 °C for 24 h in a conventional oven prior to mixing. Blends made from PLA and PBAT (Table 1) were prepared using an internal mixer with counter-rotating rotors (Haake Reomix^®^ 600, Thermo Scientific™, Waltham, MA, USA). The temperature for the heating chamber was 190 °C. Each formulation was produced in 50 g batches at a mixing speed of 30 rpm for 5 min. Finally, each batch formulation was pelletized, and compression molded specimens were made for further characterization.

Then, 1200 g of PLA were blended with 800 g of PBAT and this blend was extruded in a single screw extruder (Micro 27GL/32D, Leistritz AG, Berlin, Germany) with a temperature profile from the feed zone to the nozzle of 150/160/170/170/175/175/180/190/165 °C and a feeding rate of 1.82 g/min (screw speed 30 rpm). A three-hole nozzle was used. The diameter of each hole was 3.0 mm. Filaments obtained were pelletized to a diameter of less than 1.5 mm and stored in plastic bags until use.

Fibers were made by extrusion of the components: Pelletized PLA60 and CEO or glycerol (G) as reference plasticizer. For the extrusion, a twin-screw extruder (Process 11, Thermo Scientific™, Waltham, MA, USA) with a temperature profile of 160/160/170/180/190/190/160 °C and a single outlet nozzle of 2.5 mm was used. Finally, the fibers were cooled in water. The 60/40 pellets were dried at 60 °C for 24 h in a conventional oven prior to extrusion. A peristaltic pump (MasterFlex C/L, Cole-Parmer, Vernon Hills, IL, USA) was used for the addition of the CEO in the second port of the extruder. Three formulations were extruded: PLA60 (100%) corresponding to FC, PLA60/G (95.7%/4.3%) corresponding to FG and a formulation containing CEO, and PLA60/CEO (93.9%/6.1%) corresponding to F3. To obtain similar fiber diameters for the formulations, a take-off unit (Haake Fisons FP 1, Thermo Scientific™, Waltham, MA, USA) was used (Table 2).

### 2.3. Scanning Electron Microscopy (SEM)

From the compression molded specimens, scanning electron microscopy of cross sections for the PLA and PBAT, their blends, and the fiber specimens was performed. The specimens were fractured under cryogenic conditions using liquid nitrogen. Then, the fracture surfaces were coated with gold. An electronic microscope (Tabletop Microscope TM-1000, Tokyo, Hitachi, Japan) with an acceleration voltage of 15 kV was used.

### 2.4. Fourier Transform Infrared Spectroscopy–Attenuated Total Reflection (FTIR-ATR)

The FTIR-ATR spectra of PLA100, PBAT100, polymer blends, and the fibers were recorded using a Horiba Jobin Yvon spectrometer (Horiba, model IR2, Kyoto, Japan), equipped with an attenuated total reflection (ATR) diamond crystal. Resolution used was 4 cm^−1^ for a wavenumber range of 4000–400 cm^−1^ with 32 scans.

### 2.5. Differential Scanning Calorimetry (DSC)

PLA, PBAT, their blends, and fibers were subjected to differential scanning calorimetry (DSC Discovery Series, TA Instruments, Wakefield, MA, USA). The DSC run was: First, the sample was stabilized to 25 °C. Second, it was heated from room temperature to 210 °C at 10 °C min^−1^ and held at 210 °C for 3 min in order to erase the previous thermal history. Then, the sample was cooled to 25 °C at 10 °C min^−1^, held at this temperature for 1 min, heated again to 210 °C, and finally to room temperature at 10 °C min^−1^. Sample weight was 4.0 ± 1.0 mg. The percentage of crystallinity was calculated from the second heating using Equation (1):

(1) $$X_{c}\;\left(\%\right)\;=\;\frac{100\times \Delta H_{m}}{\left(f\times \Delta H_{m}^{0}\right)}$$

where Δ*H_m_* is the enthalpy of fusion (J/g), *f* is the weight fraction of PLA or PBAT, and Δ*H_m_*^0^ is the theoretical enthalpy of fusion for a 100% crystalline polymer, 93 J/g for PLA and 114 J/g for PBAT [31].

### 2.6. Mechanical Properties

Plaques from the PLA60 blend, 13 cm (length) × 13 cm (width) and 3 mm thickness, were compression molded in a press at 190 °C (Polystat 200 T, Schwabenthan, Wustermark, Germany) and cut (Guian Gn640MS laser cutter, Jinan, China) to obtain test specimens for mechanical properties measurement. They were subjected to tensile (ASTM D638-03) test using a universal testing machine (Instron 3345, Horsham, PA, USA). A load cell of 1 kN and tested at a speed of 1 mm min^−1^ was used. Moreover, the fibers were subjected to tensile (ASTM D638-03) tests. A 10 cm specimen was used with a load cell of 1 kN and at a speed of 5 mm min^−1^. Ten specimens were tested. All measurements were carried out at room temperature (20 ± 1 °C). Values for Young’s modulus, tensile strength, elongation, and tenacity were obtained from the machine Bluehill^®^3 software.

### 2.7. Fiber Hydrolytic Degradation

First, the viscosity and viscosimetric molecular weight for the homopolymers PLA100 and PBAT100 were determined. Chloroform (CHCl_3_) was used as a solvent. Diluted solutions of PLA/CHCl_3_ and PBAT/CHCl_3_ of 0.8%, 0.6%, 0.4%, and 0.2% m/v starting from a stock solution of 1% m/v were prepared. The intrinsic viscosity [*η*] was obtained by capillary viscometry. From the obtained value of viscosity, the viscosimetric molecular weight (*Mv*) of the PLA was determined by applying the Mark-Howink-Sakurada equation:

*η* = *K* × *Mv^a^* (2)

using *a* and *K* values reported in the literature [32,33].

Because CHCl_3_ is a common solvent for the fibers obtained from both homopolymers, the variation in the molecular weight *Mv* for the PLA100 and PBAT100 in the blends could not be monitored. However, through the variation of the viscosity in the samples studied, it can be inferred how the experimental conditions of the hydrolytic degradation (pH and temperature) influence the process at the exposure times.

The hydrolytic degradation was followed for PLA100, PBAT100, FC, and F3 fibers. On the other hand, from the morphology it was observed that the FG fiber showed porosities and cracks; therefore, the hydrolytic degradation of this fiber was not relevant.

The fibers subjected to the degradative process were weighed on an Adventurer OHAUS analytical balance, with an accuracy of ±0.0001 g. An Ubbelohde–Schott–Geräte capillary viscometer, capillary #I (capillary diameter: 0.63 mm), type 501 10/I (TA Instruments, Wakefield, MA, USA) was used to measure the molecular weight of PLA100 and PBAT100. A digital pH 11 Meter Kit Model # 35614-80 (Oakton Instruments, Vernon Hills, IL, USA) was used to monitor the pH changes of the degradation medium and for buffers’ preparation. The morphological characterization was carried out using a JEOL JSM6390 scanning electron microscope (SEM). A cryogenic fracture was done and prior to SEM observation, the fibers were gold-coated using a Blazers-SCD-030 sputter-coater (Angstrom Engineering, Kitchener, ON, Canada).

The procedure of the hydrolytic degradation of the fibers was as follows: 5 mL of buffer solution containing the fibers were centrifuged at three pH values (acid, neutral, and basic as shown in Table 3. The sample weight/volume buffer ratio was adequate to simulate the real degradation conditions of the fibers. For the experimental design, the buffer was not removed. The degradation time was 4, 8, and 12 weeks at room temperature (25 ± 1 °C) and in a thermostatic bath at 37 ± 1 °C. At the end of the experiment, the pH of the degradative medium was measured, and the mass of the fibers was verified, according to the following equation [34]:

(3) $$\Delta M\%=\;\frac{M_{i}-M_{f}}{M_{i}}\times 100$$

where Δ*M* is the weight loss variation, *M_i_* is the mass of the fibers before hydrolytic degradation, and *Mf* is the mass of the fibers at the different removal times for the different conditions.

### 2.8. Statistical Analysis

The data obtained from the characterization were analyzed using the values of the mean ± standard deviation. To establish differences between formulations, analysis of variance was performed with Tukey’s mean comparison test (*p* ≤ 0.05). For data analysis, InfoStat version 2016 software (National University of Córdoba, Argentine) was used. For hydrolytic degradation, a statistical analysis of the t-student type was made for the validation of the average results obtained.

## 3. Results and Discussion

After blend characterization, the PLA60 formulation was selected for fiber processing because it had the best relationship between strength, flexibility, and processability during the extrusion process. In relation to the DSC results (see Section 3.3), this blend was selected because showed the lowest melting peak value and also because its crystallization temperature was intermediate between values reached for the other blends, which guarantees that it can solidify quickly, which in turn is favorable for the subsequent flexibility and extrusion process of the fibers. Additionally, it had a percentage (%) of essential oil that allows an effective plasticizing effect as seen for the mechanical properties in Section 3.4.

### 3.1. Scanning Electron Microscopy (SEM)

**Figure 1:**
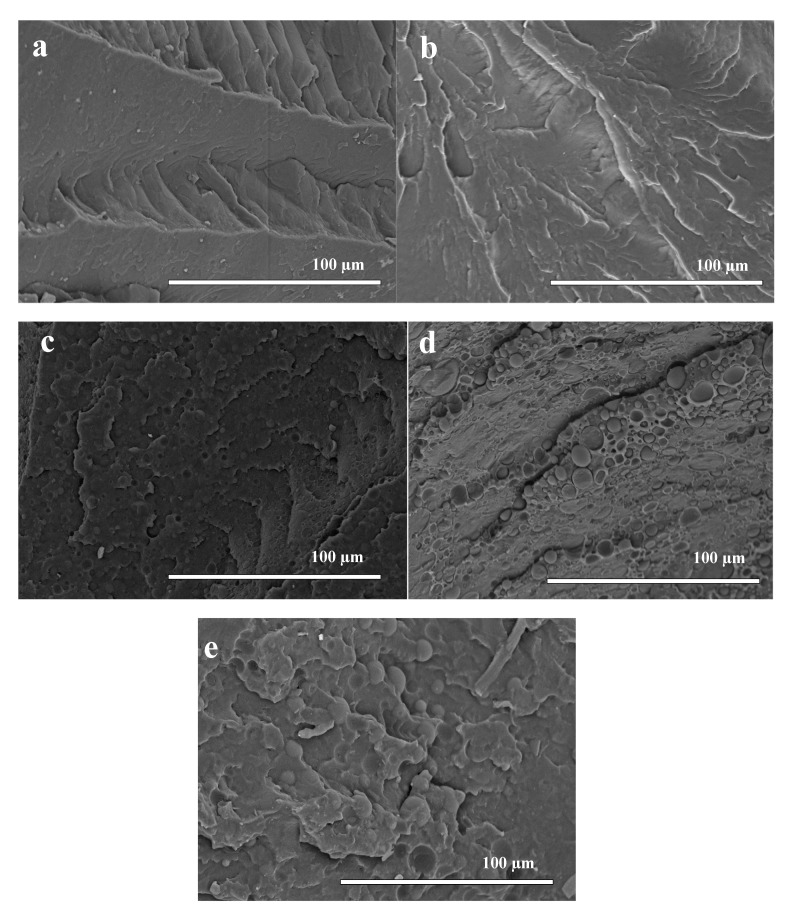
SEM micrographs (cryogenic fracture surface) of the cross-section for the homopolymers and their blends: (**a**) PLA100, (**b**) PBAT100, (**c**) PLA70, (**d**) PLA60, (**e**) PLA50.

SEM micrographs of homopolymers and their blends are shown in Figure 1. Compared to the homopolymers, PLA100 and PBAT100, in Figure 1a,b, a change in morphology is observed for the blends. As can be seen in Figure 1c, the surface is more irregular, with a clearly two-phase morphology, being PLA and PBAT the hard and the soft phase, respectively. According to the basic theory of dispersion, a surface tension is generated between the phases, which are mixed during the extrusion process. Then, the dispersed phase, which is the minority phase (PBAT), is distributed homogeneously through the matrix (PLA), maintaining some interfacial tension [35]. After cryogenic fracture, the disperse phase (PBAT) was observed in the form of spherical particles that leave some pores in the PLA matrix because of incompatibility between the two phases, which has been demonstrated in related papers of compatibility/miscibility in PLA/PBAT polymer blends [36,37].

For PLA60 in Figure 1d and PLA50 in Figure 1e, the presence of spherical particles of the disperse phase of PBAT (whose size increased as its concentration was increased) in the PLA matrix was visible. In SEM micrographs for blends with PLA and PBAT in the research of Quero et al. [38], a similar behavior was observed, which, as mentioned above, is due to the partial compatibility of the polymer phases in the blend [39,40]. It has been found in the literature that miscibility between PLA and PBAT is limited due to the differences in the solubility parameters of PLA and PBAT; therefore, there are weak interactions between polymers in the blends. Regarding the chemical structure of the polymers, PBAT have longer repeating units and more flexibility compared to PLA; thus, low interfacial adhesion and phase separation is observed [41,42]. However, the PLA/PBAT blends have been studied extensively due to a higher compatibility between PLA and PBAT compared to PLA/PCL and other polyesters blends [43]. Therefore, in order to improve the interfacial adhesion, plasticizers can be used [41].

**Figure 2:**
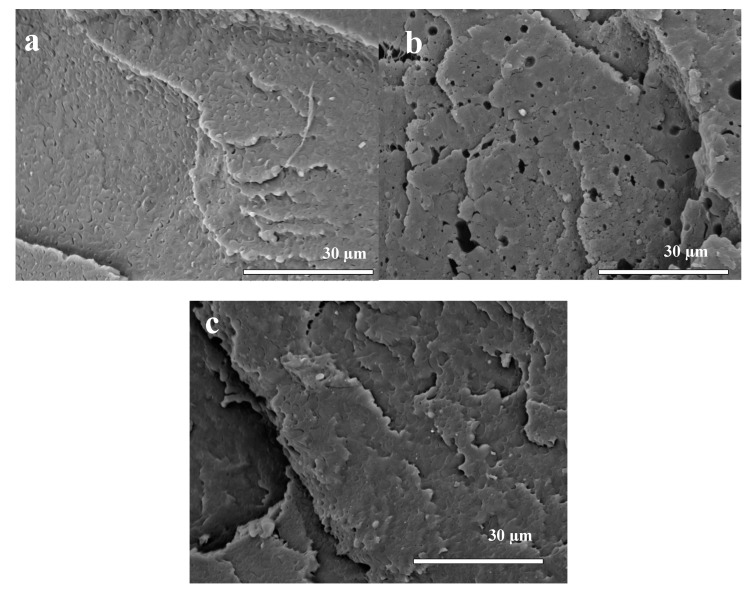
SEM micrographs of the (cryogenic fracture surface) of the cross-section for the bio-based polymer fibers: (**a**) FC, (**b**) FG, and (**c**) F3.

In Figure 2, the morphology of the cross-section of the fibers can be seen. When the PLA blend was double processed for the preparation of the FC fiber, the morphological differences between the two phases were remarkably reduced, as seen in Figure 2a. The PLA and PBAT phases were more homogeneously distributed over the entire observed area. From Figure 2b it is observed that the FG fiber presented porosities and cracks. For the micrographs of the polymeric fiber with CEO (F3) in Figure 2c, no significant changes were observed in the structure of the material with the incorporation of the essential oil. A surface with uniform distribution of the polymers without pores is shown. Therefore, the use of CEO has a clear advantage as a plasticizer compared to glycerol because it does not create porosity or cracks in the blends.

### 3.2. Fourier Transform Infrared Spectroscopy–Attenuated Total Reflection (FTIR- ATR)

**Figure 3:**
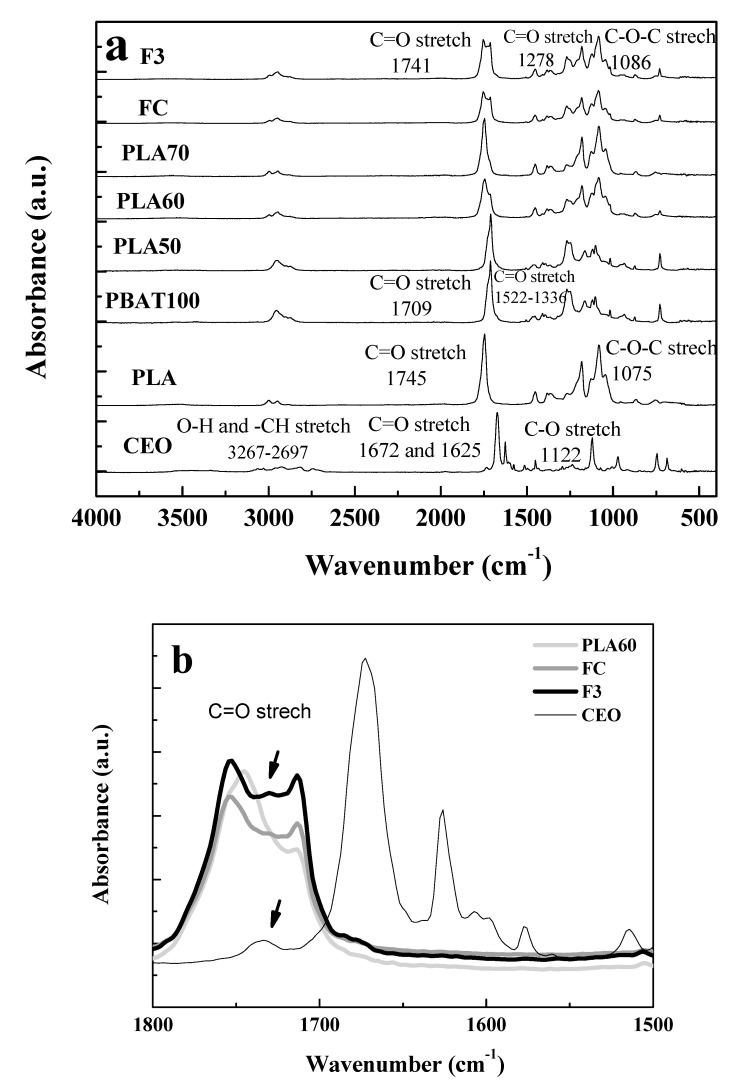
FTIR spectra of: (**a**) CEO, homopolymers (PLA100, PBAT100), blends (PLA50, PLA60, PLA70), and fibers (FC, F3) and (**b**) detail for CEO, PLA60, FC, and F3.

The FTIR spectra for the essential oil, homopolymers, the blends, and the fibers are shown in Figure 3.

To understand the interactions between the CEO and the polymer blends, FTIR measurement was done. The main characteristic absorption bands for CEO, PLA100, and PBAT 100 are shown in Table 4.

In previous studies, it was found that cinnamaldehyde was the majority component for the CEO, a phenolic terpenoid that possesses antimicrobial activity against different microorganisms [49,50]. Regarding the PLA50, PLA60, and PLA70 blends, it can be seen that the main peaks are the addition of the homopolymers’ peaks (PLA100 and PBAT100) and only small differences were found for the peaks at around 1730 cm^−1^ and at 1262 cm^−1^, which are related to C=O stretching. The peak around 1730 cm^−1^ was shifted depending on PLA content in the blend being the change more noticeable from PLA50 to PLA60. For PLA50 (PBAT content 50%), the peak value was near PBAT100 compared to PLA70 (PBAT content 30%) with the peak position near PLA100. For PLA60, the peak value was between PLA100 and PBAT100. On the other hand, the peak at 1262 cm^−1^ was also between PLA100 and PBAT for PLA50 and PLA70 and decreased for PLA60. Some authors have reported a possible interaction of the carbonyl group between PLA and PBAT [43,51], leading to partial miscibility of the blends. This was seen specially in the SEM micrographs of the fibers after a second processing in which the miscibility of the blends was improved and a more homogeneous surface was observed. Also, the enhancement of the mechanical properties which will be discussed in Section 3.4.

Because of the low concentration of CEO (6.1%), FTIR spectra for FC and F3 were similar, as can be observed in Figure 3b. However, there were some small differences for the absorption band related to C=O. F3 is the fiber resulting from the incorporation of CEO to PLA60 and FC is the fiber extruded from PLA60 formulation. After the PLA60 extrusion to form the FC fiber, the peak at around 1750 cm^−1^ corresponding to C=O stretching was broadened. It is well known from the literature, that PLA, during processing, suffers thermo-oxidative degradation due to different factors such as temperature, oxygen, and mechanical shear, with the subsequent formation of ester, carbonyl, or carboxyl groups. It can occur when the polymer is extruded at around 200 °C near the melting point or below [52]. Therefore, the main changes in the characteristic absorption bands for the FTIR spectra occurred at 1750 cm^−1^ due to stretching of carbonyl group demonstrating the possible chain scission of PLA at around 200 °C [53].

On the other hand, in Figure 3b, it can be seen that for F3 there is a small shoulder (arrowed), which is also seen for the CEO at the wavelength of 1732 cm^−1^. The presence of this shoulder could be indicative of the incorporation of CEO into F3 fiber after blending with PLA60 [54]. However, regarding the stability of essential oils, their components are susceptible to oxidation, chemical transformation, or polymerization [55].

### 3.3. Differential Scanning Calorimetry (DSC)

**Figure 4:**
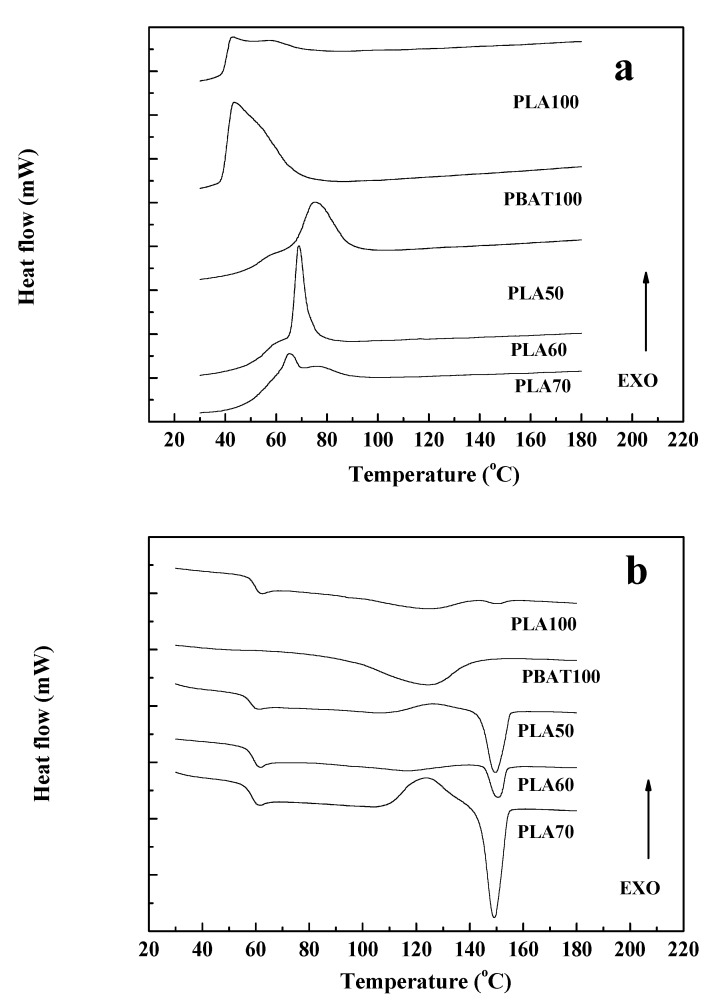
DSC thermograms of homopolymers and their blends: (**a**) Cooling and (**b**) second heating.

**Figure 5:**
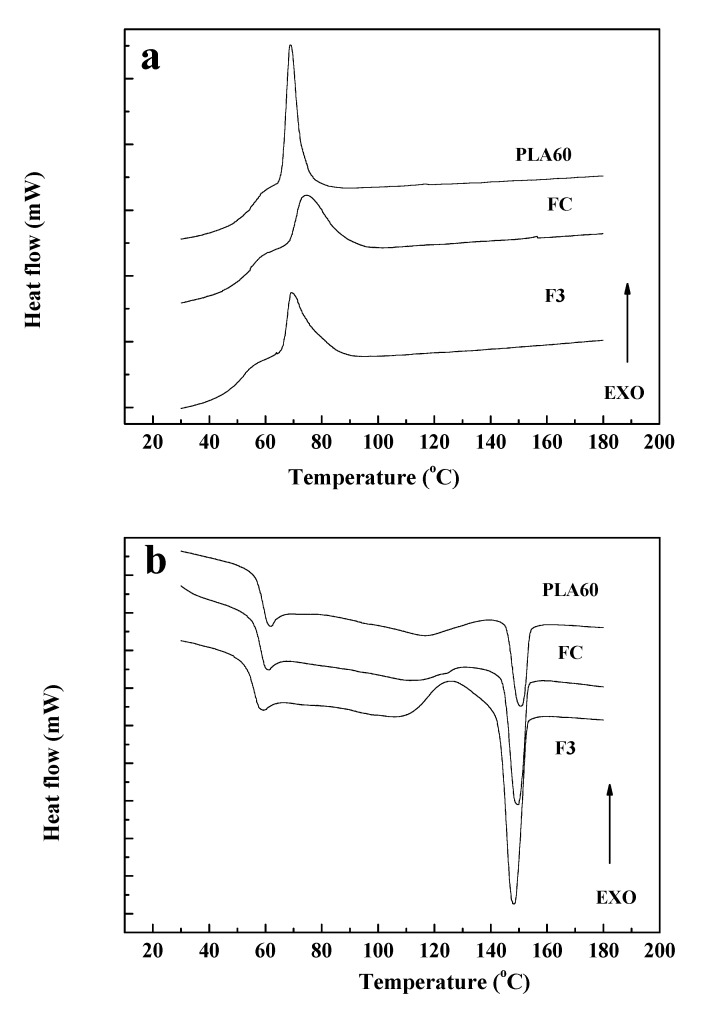
DSC thermograms for PLA60 and bio-based polymer fibers FC and F3: (**a**) Cooling and (**b**) second heating.

The DSC thermograms for the controlled cooling of the homopolymers, PLA/PBAT blends, and fibers (FC, F3) after a first heating step to erase their previous thermal history and for the second heating after inducing controlled crystallization at a rate speed of 10 °C/min are shown in Figure 4 and Figure 5. Similar values of T_g_, T_c_, T_m_, enthalpy, and crystallinity for PLA, PBAT, and PLA60 were obtained for composite fibers of PLA, PBAT, and pine essential oil [10].

In Figure 4a, from the cooling thermograms, the crystallization temperature (T_c_) of PLA100 and PBAT100 was similar (43 °C). In the literature, reported values for the T_c_ of PLA are 93.8 °C [56] and 100 °C [57]; and for PBAT 86.8 °C; 83.1 °C [58] and 70.7 °C [59]. As seen from the results, the T_c_ (43 °C) was lower for PLA and PBAT compared to the values reported in the literature. This could be due to reprocessing and possible thermal degradation of PLA during processing as mentioned before [60].

For the blends, T_c_ was increased, being 65 °C for PLA70, 68.7 °C for PLA60, and 75.1 °C for PLA50. This suggests that the higher the PBAT content in the sample, the higher the starting temperature for the PLA crystallization process [61], which could be indicative of partial miscibility between polymeric phases [43].

During crystallization, it can be seen if the polymer was able to crystallize or not. If not, this is reflected as cold crystallization, which appears in the second heating for fusion as reported in the literature by several authors [62,63,64,65]. In Figure 4a, it is observed for PLA 70 that the curve of crystallization is not as narrow for PLA 60. Therefore, during the second heating, PLA 70 showed the broadest cold crystallization range of 120–135 °C, as seen in Figure 4b.

For the second heating, in Figure 4b, PLA100 had a glass transition temperature (T_g_) of 62.4 °C. Also, an endothermic cold crystallization peak (T_cc_) at 124.6 °C and an endothermic melting point (T_m_) at 150.3 °C were observed. For the PBAT100, an endothermic peak was found at 124.1 °C, corresponding to the T_m_. Although the T_g_ of PBAT was not determined experimentally, a value of −34 °C is reported in the literature [55]. PLA60 and PLA50 blends showed three transition temperatures. A first thermal transition (61.9 and 61.4 °C, PLA60 and PLA50, respectively) for the T_g_ of PLA, a second exothermic peak (116.8 and 126.0 °C, PLA60 and PLA50, respectively) for the T_m_ of the PBAT, and finally a second endothermic peak (150.7 and 149.5 °C, PLA60 and PLA50, respectively), for the T_m_ of the PLA. Signori et al. [66] reported that the exothermic peak for the T_m_ of the PBAT is related to a reorganization of the amorphous crystalline domains for the blends due to the increase in temperature leading to the phenomena of cold crystallization. For the PLA70 and PLA50 blends in Figure 4b, cold crystallization was observed. Also, it was observed to a lesser extend for PLA60 at the same temperature range as for the T_m_ of PBAT.

The modification of the thermal transitions in the fibers with the presence of the oil in the PLA60 formulation can be seen in Table 5.

During the cooling process, it was observed in Figure 5a that the crystallization range was broadened for the fibers (FC and F3) compared to PLA60. From Table 5, comparing the PLA60 blend with the FC fiber, the T_c_ started at higher temperature and the percentage of crystallinity decreased, perhaps because reprocessing improved partial miscibility of both phases [67] or by thermal degradation due to the second extrusion process [68].

After the second heating, F3, showed an exothermic peak above 125 °C due to a crystallization induced by heating as seen in Figure 5b. However, this peak was not seen for FC. Also, the T_c_ (onset) for F3 appeared earlier than for Fc and PLA60, because the oil in its plasticizing action allows the molecular rearrangement compared to the samples without CEO. Also, for the second heating in Figure 5b, it is seen that the crystallization process is completed in more extend for F3 through a process of cold crystallization than for the other samples, as indicative of CEO plasticizing effect [69,70].

The displacements in the T_g_ and T_m_ of the fibers (FC and F3) with respect to the transition temperatures of the homopolymers as seen in Figure 5b, can be attributed to the double processing of the material to obtain the fibers and to the presence of the CEO in the fiber as a plasticizer. The change in crystallinity due to re-processing, possibly induced by orientation during extrusion, is observed in the FTIR spectra from Figure 3a with a shifting to a lower value of wavelength for the C=O peak (1730 cm^−1^). This is in agreement with the endothermic fusion peak that is more intense for F3, followed by FC and PLA60 as seen in Figure 5b for DSC in correlation with the change in peak intensity of the carbonyl group from the FTIR results.

Related to the percentage of crystallinity, in Table 5, this percentage is approximately the same for all samples. For the homopolymers, PLA100 and PBAT100 had a similar percentage of crystallinity as demonstrated by other authors [36,71]. Samples for DSC were taken from the compressed plaques, which were previously subjected to a thermal process. For PLA50 and PLA60, the percentage of crystallinity was also comparable. However, for PLA70 and PLA100, this percentage is higher. The chemical structure of PBAT has benzene rings [72] that restrict PLA mobility and structural regularity, impeding the reorganization of PLA chains; thus, the percentage of crystallinity is reduced as PBAT content is increased in the blends. Moreover, the cold crystallization peak is larger for the blends than for PLA100, as can be seen for PLA70 as compared to PLA in Figure 4b [73].

Although the higher content of PBAT in the blend accelerate the crystallization rate process for PLA, which started at a higher temperature, as aforementioned, it had little effect on the final percentage of crystallinity for the different blend compositions. Therefore, crystallinity of the blends with the incorporation of PBAT into the PLA matrix did not change significantly [74].

On the other hand, from Table 5, it is observed that the T_c_ of FC had a slight increase (6.1 °C) with respect to PLA60, while that of F3 was similar. This change in the T_c_ of FC, as mentioned above, would be due to the double processing suffered by the material during the preparation of the fiber [51].

### 3.4. Mechanical Properties

Results for tensile mechanical test can be seen in Table 6. The addition of PBAT to the blends modified the mechanical properties of PLA100. It was observed that as the percentage of PBAT in the blends increased, a lower strength (MPa) was required to reach similar percentages of elongation. However, comparing PLA50 with PBAT, PLA50 is composed of 50% PLA and 50% PBAT, its Young’s modulus value was 232.2 ± 20.0 MPa having PBAT a Young’s modulus value of 1.6 ± 0.2 MPa. From the thermal properties (Table 5), the T_g_ value for PBAT was −34.0 °C and for PLA50 it was 61.4 °C. When the T_g_ value is below room temperature, the polymer is in a rubbery state and then, the value of Young’s modulus is lower compared to a polymer, of which the T_g_ value is above room temperature with a fragile behavior and high modulus [75].

The values of Young’s modulus and tensile strength at break, for tensile tests of homopolymers and blends, showed statistically significant differences. From the results, it can be seen that Young’s modulus is modified by the presence of PBAT in the PLA matrix, which is indicative of partial miscibility between the two polymeric phases [32].

On the other hand, the elongation at break increased from 8.8% (PLA100) to 17.9% for PLA60 and 27.4% for PLA50. PBAT100 reached an elongation near to 1200%. Although elongation at break and tenacity did not show significant statistical differences, there was a slight increase in the value of elongation at break for the blends, as content of PBAT increased, due to the higher flexibility of this polymer compared to PLA [31,76].

The increase of elongation at break with the incorporation of PBAT into PLA blends, had been reported by some authors [36,42]. They have found that upon the addition of 20% PBAT to PLA/PBAT blends, there was an increase in the percentage of elongation at break of 26.6% [39] and 257% [33]. In the literature, Ecoflex^®^ is reported as a strong and flexible material with mechanical properties like low-density polyethylene (LDPE). As a result, the obtained products are tear-resistant and flexible [77]. Therefore, the elongation at break was enhanced due to the incorporation of PBAT into the blend.

“In polymer blends, the mechanical properties are affected by blend composition and miscibility between phases” [78,79]; in this case, PLA and PBAT as main components. For the PLA60 blend, it had a lower average value of elastic modulus (E) compared to the values of the PLA matrix and the dispersed phase (PBAT), which is a positive aspect. Additionally, the tensile strength had a low value. However, this value is due to the flexibility, which is reflected in the deformation at the break reached. As evidenced by DSC, for this blend, in the molten state, the phases have a certain compatibility that exerts an influence in terms of the crystals that are formed with consequences in the observed mechanical response. However, the decrease in the mechanical properties, especially for Young’s modulus and tensile strength at break in the blends due to PBAT incorporation, can be attributed to the partial miscibility between PLA and PBAT, rather than the change in crystallinity for PLA [8], as mentioned before.

Table 6 shows the results for the tensile properties of the fibers. When PLA60 blend undergoes double extrusion processing to obtain the control fiber (FC), the mechanical properties changed, reflected in a 600% increase in Young’s modulus for the tensile tests. It is observed that there was an increase in the Young’s modulus and a decrease in the elongation at break for the fibers (F3 and FC) compared to the blend compositions and homopolymers. It has been reported that an increase in Young’s modulus and tensile stress and a decrease in elongation at break is related to the induced orientation in the polymer chains during processing [51,80]. Although it is expected that the incorporation of essential oil into a polymer matrix tends to decrease the Young’s modulus, the tensile strength and to increase the elongation at break, in this case, an increase in Young’s modulus for F3 compared to FC was observed due to the induced crystallization during heating as evidenced by DSC. The increase in Young’s modulus was an indicative of a higher resistance to deformation of the sample. This could be due to carbonyl group interaction between the PLA/PBAT as mentioned in the FTIR measurements. Moreover, the higher value of elongation at break for the F3 fiber compared to FC is related to the CEO plasticizing effect as evidenced by the DSC results; then, influencing the mechanical behavior of this fiber [54,81].

As seen in Table 6, there were no significant differences for the fibers when adding CEO (F3) to the control FC fiber. On the other hand, similar behavior was observed for Young’s modulus, tensile strength at break, and elongation at break, in which the formulations did not show significant statistical differences, except for the case of fiber with glycerol (FG) for Young’s modulus and tensile strength at break. However, the difference in the plasticizing effect between the CEO and the glycerol [82] was not significant enough to be reflected in the elongation at break. The decreased in mechanical properties for FG is evidenced in Figure 2b with the presence of microcracks and porosities observed in the fiber surface due to possible migration of the plasticizer [83,84].

**Figure 6:**
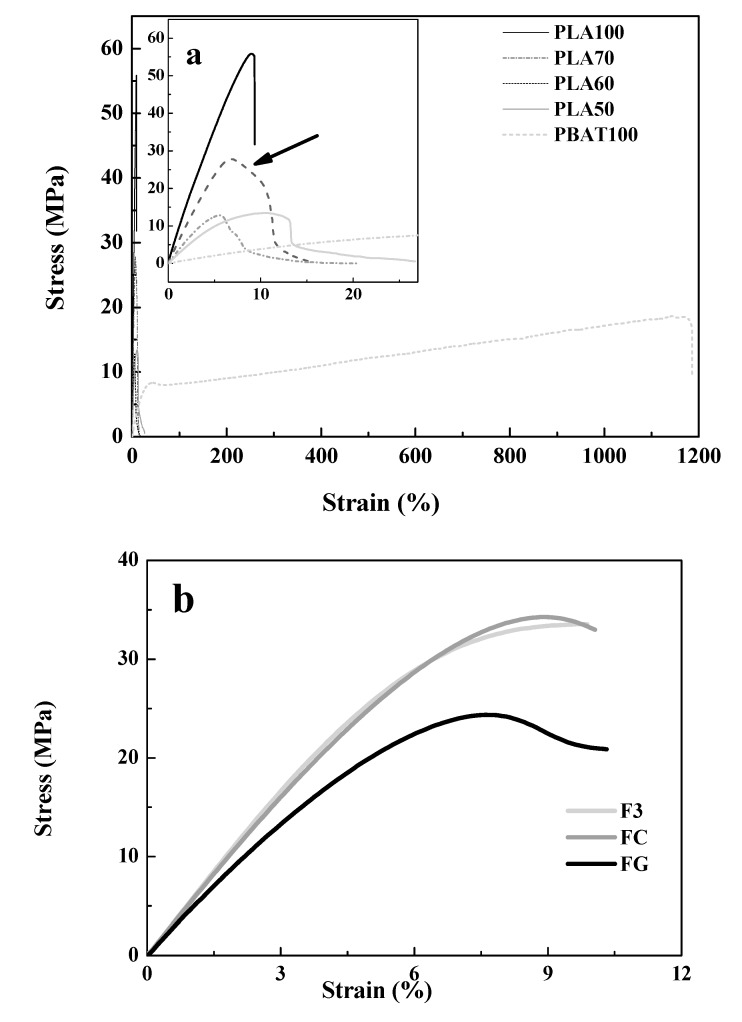
Stress–strain curves of: (**a**) Homopolymers and their blends and (**b**) bio-based polymer fibers.

In Figure 6, a representative stress–strain curve for the homopolymers and blends is seen. In Figure 6a, a detail of the curve was added to better observe the mechanical behavior of PLA and the blends in the 0%–30% elongation region. Yield deformation can be observed (arrowed) due to plastic deformation of PBAT [85,86]. In Figure 6b, the stress–strain curve of the fibers is shown.

It can be seen in the detail of Figure 6a that PLA100 exhibited a brittle fracture without plastic deformation. On the other hand, PBAT showed a ductile fracture. For the blends, the brittle fracture slightly changes to ductile due to addition of PBAT to PLA [87]. Only a small change is observed due to phase separation and partial miscibility of the components of the blends, as mentioned above. The elongation at break increased with PBAT content as observed in the insert of Figure 6a and values reported in Table 6 (from 14.8% for PLA70 to 27.4% for PLA50).

On the other hand, the tenacity was calculated from the stress–strain curve. Data are shown in Table 6. For the blends, no statistically significant differences were observed for the tenacity; the only difference that was found was with respect to PBAT (Table 6).

From Table 6 and Figure 6b, it was observed that as CEO was incorporated into the fiber, the tenacity was improved. The FC control fiber and the fiber with glycerol FG had lower tenacity values compared to F3. Statistical differences were found among fibers due to the addition and type of plasticizing (glycerol or CEO) [88]. The modification of the tenacity of a polymer like PLA with another biodegradable polymer with higher tenacity and its compatibilization, will promote miscibility [63,89]. From the point of view of manufacturing is important to consider that the final application will depend on mechanical properties: Young’s modulus, elongation, and tenacity.

### 3.5. Fiber Hydrolytic Degradation

In the case of the hydrolytically degraded samples, the results of the fibers exposed to the degradative medium during a period of 12 weeks at a temperature of 25 °C were considered, because this is the test condition that is closest to the conditions of use of the fibers. On the other hand, the degradation process was also carried out at a temperature of 37 °C in order to simulate hot and humid environments in which an accelerated degradation process could occur. Therefore, the results obtained for the PLA100 fiber were considered since these fibers showed evidence of degradation under alkaline pH test conditions will be discussed later. The rest of the samples (PBAT100, FC, F3) did not present conclusive evidence of degradation at the surface level and they were not included.

The influence of variables such as the chemical structure of the polymer, the characteristics of the degradation medium (temperature and pH), and the duration of the hydrolysis were considered for the analysis of the results [90]. Related to the mechanisms of biodegradation, this was a purely hydrolytic process, particularly the random fragmentation of the macromolecular chains that took place. Thus, the variation in weight, molecular weight, and pH of the medium could give indications of how this process occurred and under what conditions such degradation can be more critical [91]. In heterogeneous systems, such as polymers that have heteroatoms like oxygen and unsaturation in their main chain (as is the case of the materials under study), they are intrinsically susceptible to low hydrolysis processes under the reaction conditions established in this research.

It has been found for polyesters that the hydrolytic degradation process takes place in two stages [6,92,93]. The first stage occurs preferentially in the amorphous zones, which contain chain end segments, folds, and chains not fixed with free rotation, whose structures are essentially disordered, and facilitate the diffusion and attack of the hydrolysis medium to the entire mass of molecular chains (initially in its amorphous phase). The second stage results from the attack on the crystalline zones (or blocks) of the material, which becomes effective once the amorphous zones have been seriously eroded or degraded. This last stage occurs much slower than the first, due to the highly ordered structure of the crystalline zones, which makes the diffusion of the hydrolytic medium difficult. It is also important to consider the glass transition temperature (T_g_) of each polymer involved in the elaboration of the fibers, because the thermal difference between that value of T_g_ and the testing temperature will determine if the polymer has a greater capability of getting molecular mobility of the main chain segments [94]. It has been reported in the literature that for PLA, the T_g_ is 53 °C, an endothermic cold crystallization peak (T_cc_) occurs at 124 °C, and an endothermic melting point (T_m_) is found at 150 °C [62]. For the PBAT, an endothermic peak was presented at 124.1 °C, corresponding to the T_m_, and its T_g_ is −34 °C [7,43]. Therefore, the system becomes less crystallizable and its degradation could be faster, as well as the PBAT in relation to the PLA in the composition of the blend, which is one of the objectives of this research.

“It is important to consider that when a hydrolytic degradation process begins, the diffusion process occurs first on the surface of the sample. Then, the degradative medium can diffuse to the inside. Thus, the molecular fragmentation of the molecular chains initially occurs mainly through a surface-erosion process” [95,96,97]. Therefore, changes in the weight and molecular weight distribution of the sample will take place, among other modifications [98]. From the results of capillary viscometry, the average molecular weight (*Mv*) was 17 × 10^4^ g/mol and 1.1 × 10^4^ g/mol for PLA and PBAT, respectively.

**Figure 7:**
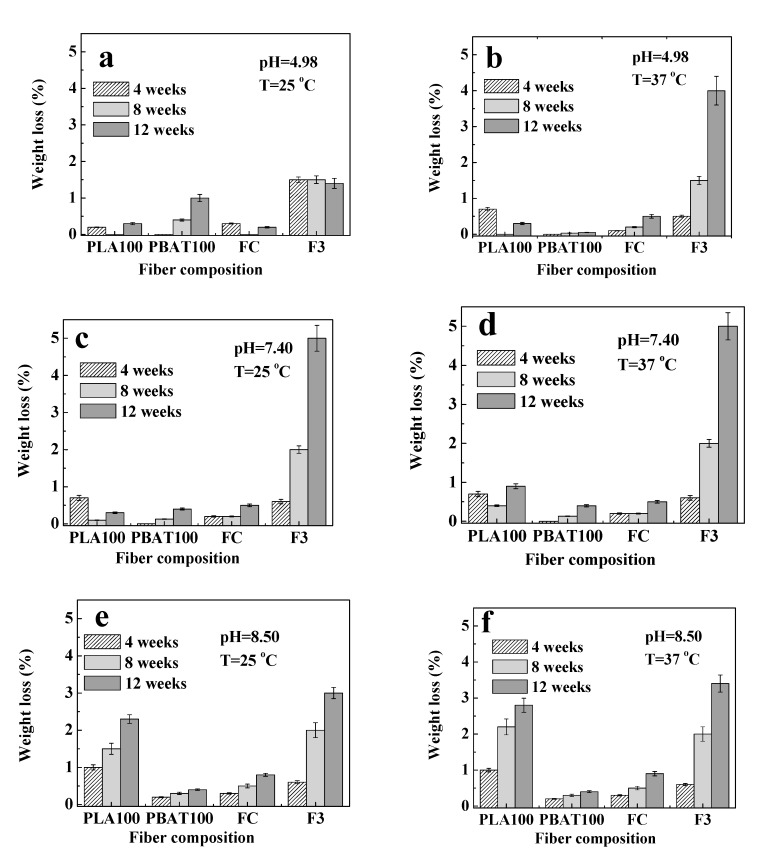
Fiber weight loss at 4, 8, and 12 weeks for: pH = 4.98 at (**a**) T = 25 °C and (**b**) T = 37 °C; pH = 7.40 at (**c**) T = 25 °C and (**d**) T = 37 °C; and pH = 8.50 at (**e**) T = 25 °C and (**f**) T = 37 °C.

As mentioned before, the weight loss is one of the variables used to analyze the behavior of the material during the period of hydrolytic degradation under study. The results obtained are shown in Figure 7. The weight loss of the fibers was below 5% during the period studied. Some authors have reported a weight loss of 27% after 16 weeks [4], 3% after 5 weeks [99], and 37% after 28 days (almost 4 weeks) [100] for in vitro hydrolytic degradation of PLA.

The most noticeable change in weight loss for the fibers during the hydrolysis process occurred at 37 °C. F3 weight loss was higher than for FC. The problem of plasticized blends due to plasticizer migration is well known in the literature [101]. The diffusion coefficient depends on type and concentration, molecular weight, compatibility, environmental conditions, among others [102,103]. It is possible that the oil can diffuse and migrate contributing to the weight loss of the fiber. Real-time release study of CEO from the fibers is in progress.

Similar results were reported by Sabino et al. in 2003 [98]. Fibers of Polyglactin 910 sutures (a polyester copolymer) degraded in buffer with pH 6 and 7 were studied. It was reported that the weight loss was associated with the chemical structure of monofilaments. The structure of Polyglactin 910 is formed by lactic acid units and glycolic acid. The lactic component gives great resistance to hydrolysis for the copolymer, due to the steric hindrance of the methyl group, (additional to that of the ester group), compared with that of glycolic acid. Therefore, as a copolymer, it has a greater percentage of amorphous zones that facilitated degradation. In a similar way, this behavior can be observed in this study for the PBAT, which has a rigid aromatic ring structure in one of its copolymeric structural units, which limits its flexibility, and therefore, it can increase its amorphous character. Moreover, in Figure 7, a small weight gain in the first weeks for PLA100 (pH = 4.98 and 7.40 for both temperatures tested) and for FC at pH = 4.98 and 25 °C was observed. This can be associated with the absorption of the solution used for hydrolysis (or its salts) and/or a recrystallization process that may occur in the system [104].

On the other hand, the final weight loss observed at week 12 can be associated with the release of degraded products from the amorphous phase, which can diffuse to the degradative medium, and which will also be responsible for possible changes in pH. Therefore, the pH measurements were done to monitor if acid type compounds (R-COOH) were released during the hydrolysis process modifying the pH value of the solution. Any change in pH if indicative that the degradation process occurs regardless of weight loss or changes in the morphology of the samples [40].

**Figure 8:**
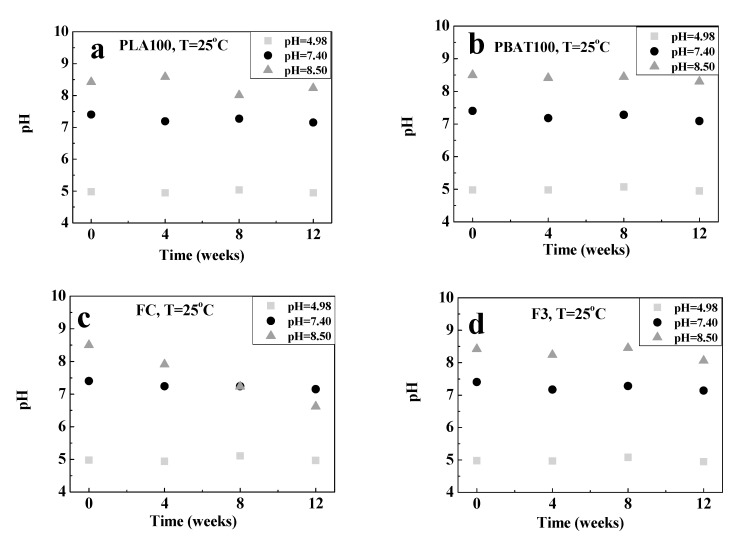
Variation in the pH of the buffer solutions used for degradation at 25 °C with time for: (**a**) PLA100, (**b**) PBAT100, (**c**) FC, and (**d**) F3.

**Figure 9:**
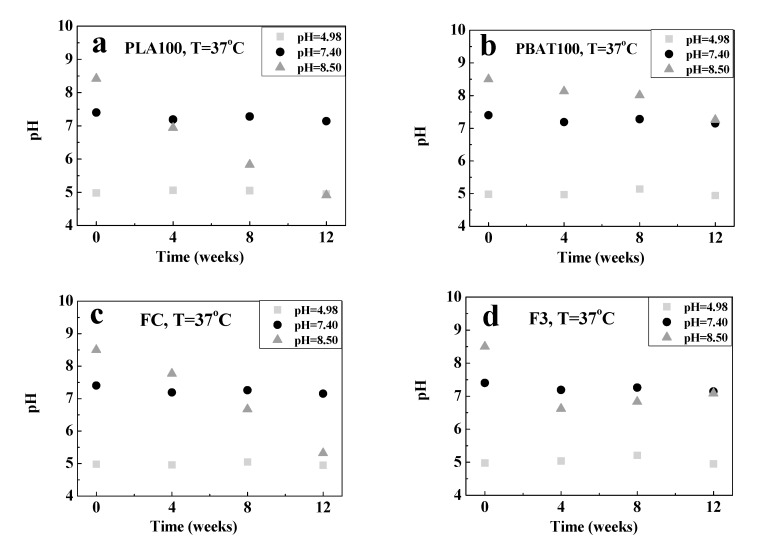
Variation in the pH of the buffer solutions used for degradation at 37 °C with time for: (**a**) PLA100, (**b**) PBAT100, (**c**) FC, and (**d**) F3.

Then, the influence of the pH variation of hydrolysis medium during the degradation process of the fibers was studied. The values obtained are shown in Figure 8 and Figure 9. For the fibers at 25 °C, the pH remains relatively constant, except for FC at pH = 8.50. However, at 37 °C, the pH of the solution decreased for all fiber compositions. This behavior is indicative of the hydrolysis reaction that was taking place. A possible explanation is the generation of low molecular weight species (oligomers having -COOH groups), which are acid species that migrate to the medium accelerating the degradation process (acid catalysis); and decreasing the pH [105].

Another possible explanation could be related to the attack on the amorphous zones due to, essentially, two factors. The first, associated with the greater susceptibility of the amorphous region to degradation, as a consequence of the empty volume that helps the process of water diffusion into the sample, and the second due to the greater mobility and lower degree of packaging of the chains that were achieved once the segments located in the amorphous zones were hydrolytically attacked [104]. Sabino et al. [92] studied the hydrolytic degradation of Poly (p-dioxanone) PPDX. It was found that the pH of the solution medium (initially with a value of 7.44) remained essentially constant until the sixth week of the hydrolytic process, from which it decreased until reaching a value of approximately 5.64. This decrease in the pH of the medium in the last weeks of the process was an indicative of the resistance of PPDX to hydrolytic degradation and can be associated with its chemical structure.

**Figure 10:**
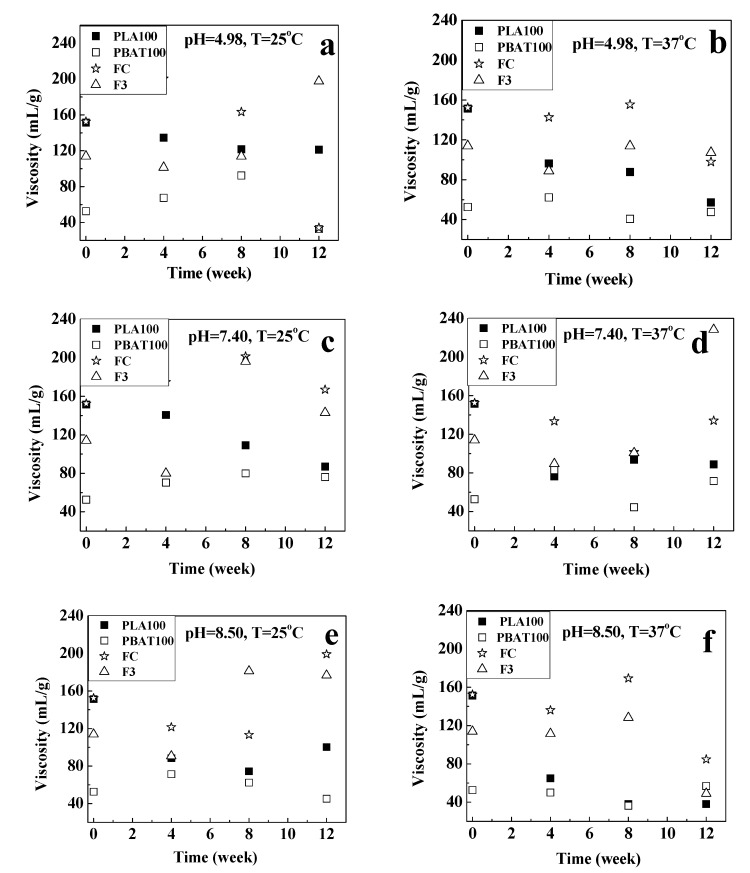
Variation in the viscosity for the fibers for: pH = 4.98 at (**a**) T = 25 °C and (**b**) T = 37 °C; pH = 7.40 at (**c**) T = 25 °C and (**d**) T = 37 °C; and pH = 8.50 at (**e**) T = 25 °C and (**f**) T = 37 °C.

The behavior observed in the gravimetric (weight loss) and pH changes in the solution medium are related to the processes of molecular fragmentation that occur during hydrolysis. This is also reflected in the viscosity changes observed in Figure 10. There was an increase in the viscosity of the solution for F3 (except for pH = 8.50 and T = 37 °C) and a decrease for FC (except for 25 °C for pH = 8.50). The increase can be attributed to the generation of a wide molecular weight distribution, and the formation of oligomers, which can generate greater molecular entanglements, extended, or branched chains, resulting in an increase in viscosity [31]. On the other hand, by week 12, at the end of the hydrolysis, the decrease in viscosity observed was related to a decrease in molecular weight. However, the viscosity of PLA100 was lower than the viscosity of the fibers (FC and F3) as indicative of polymer chain degradation [106]. For the PBAT100 fiber, the viscosity remained almost constant.

**Figure 11:**
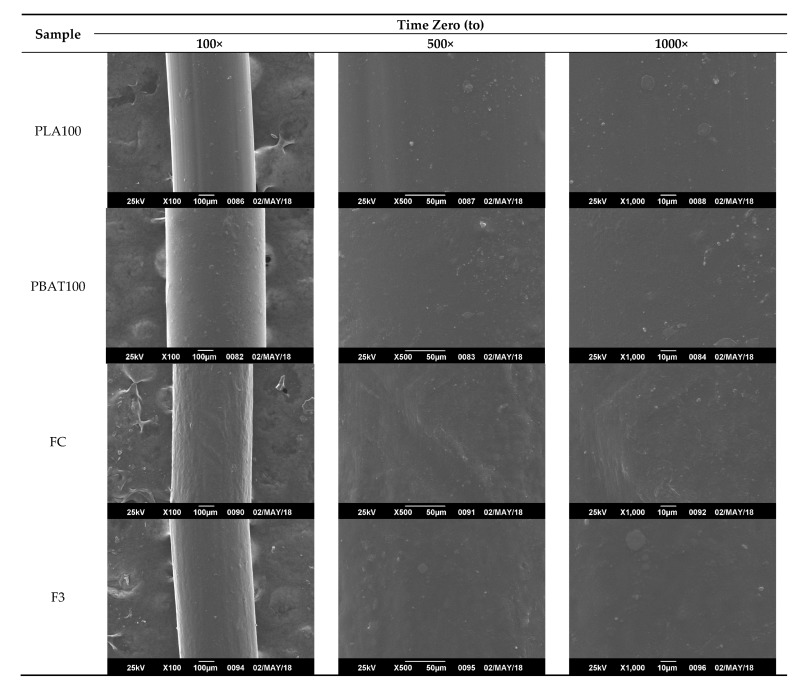
SEM micrographs of fibers’ surface morphology of the non-degraded fibers. Time zero (*t*_o_).

To observe the degradation mechanism of the fibers as a function of the hydrolysis time, they were analyzed by SEM, especially on the surface, which were in direct contact with the hydrolytic solution medium. The hydrolytic attack started on the surface and went through the interior of the fiber. As seen in Figure 11, before the degradation process, the fiber fragments showed a cylindrical shape, homogeneous, and with smooth appearance.

**Figure 12:**
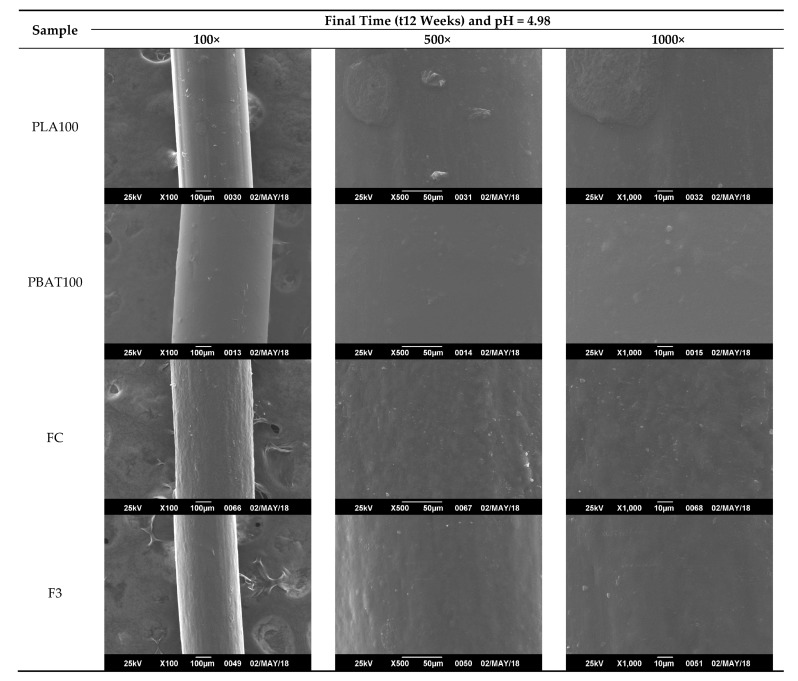
SEM micrographs of fibers’ surface morphology degraded for 12 weeks at pH = 4.98 and T = 25 °C.

**Figure 13:**
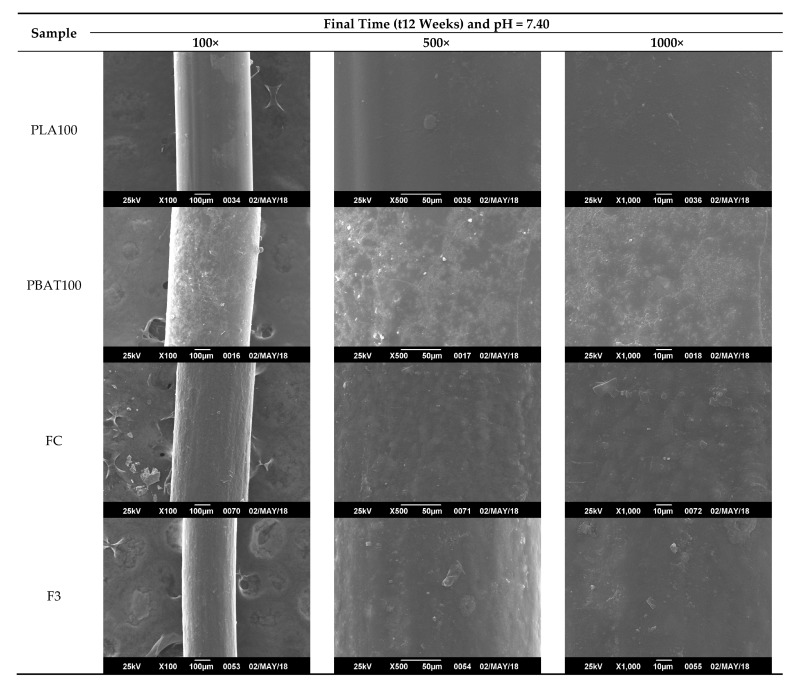
SEM micrographs of fibers’ surface morphology degraded for 12 weeks at pH = 7.40 and T = 25 °C.

**Figure 14:**
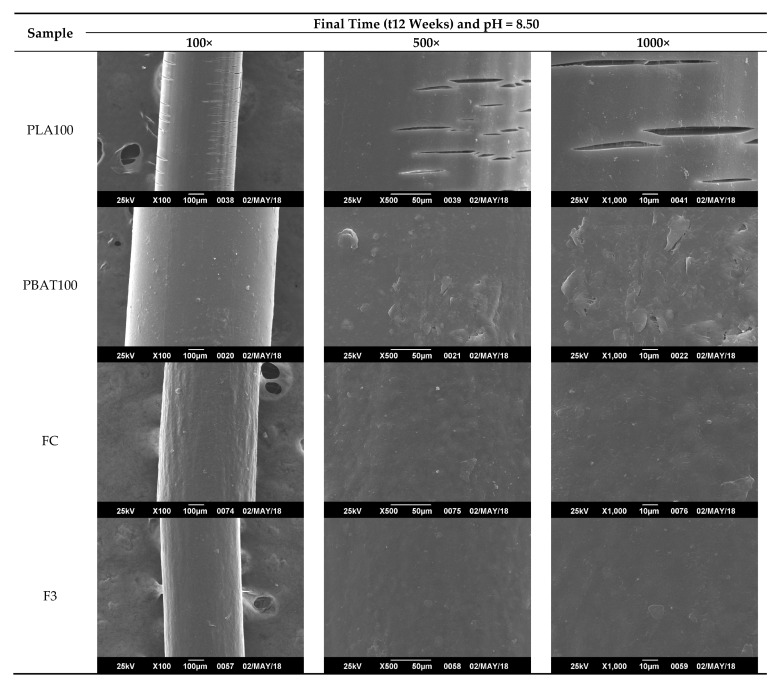
SEM micrographs of fibers’ surface morphology degraded for 12 weeks at pH = 8.50 and T = 25 °C.

From Figure 11, Figure 12, Figure 13 and Figure 14, it is observed that after a few weeks of being immersed in the hydrolysis medium, there were some remnants of salt of the buffer solution present on the surface and slight changes in roughness. Hence, only the micrographs at week 12 are shown, since in the previous weeks, important changes were not observed. For the acidic and neutral pH, this change was not significant, despite changes in the weight and final pH of the solutions. This may be due to possible release of the degradation products or polymer chains in a regular way, which cannot be seen microscopically on fiber’s surface. However, in Figure 13, for the alkaline pH solution, it seemed that a more significant degradative process on the PLA100 was achieved, in accordance with those observed for Figure 7f, Figure 8a and Figure 10f.

In Figure 13 and Figure 14, the formation of micro cracks in the fiber are observed, providing evidence of the hydrolytic degradation process. Subsequently, from these cracks, the fragmentation of the filaments occurred. These cracks were randomly distributed on the surface, propagate circumferentially around the fibers, and deepen inside as the degradation proceeded. This process took place until, eventually, the fragmentation of the fibers occurred. On the other hand, it can be seen that the degree of cracking on the surface was higher the longer the samples stayed immersed in the hydrolysis medium and alkaline pH; thus, the presence of hydroxyl species (-OH) could induce a more significant chemical attack on the electrophilic groups present in the chains of the polymer PLA [69] and not for the PBAT and blends. The presence of the oil in the F3 formulation would avoid the mentioned effect it gives the fiber a more hydrophobic character. During the first stage of the hydrolytic degradation process, the samples containing oil as plasticizer are resistant to hydrolytic degradation, as degradation progresses, the plasticizer migrates, and the hydrolytic media can diffuse inside the fibers [107]. Therefore, the hydrolytic degradation of fibers added with oils is more noticeable for longer times as evidenced in the morphological changes of the fibers.

Ndazi et al. [108] studied the hydrolytic degradation of polylactic acid/rice hulls composites in water at different temperatures. The authors observed that there was an enhancement of the disintegration during hydrolysis in water of the PLA matrix due to the incorporation of rice hulls to the composites based on SEM characterization.

**Figure 15:**
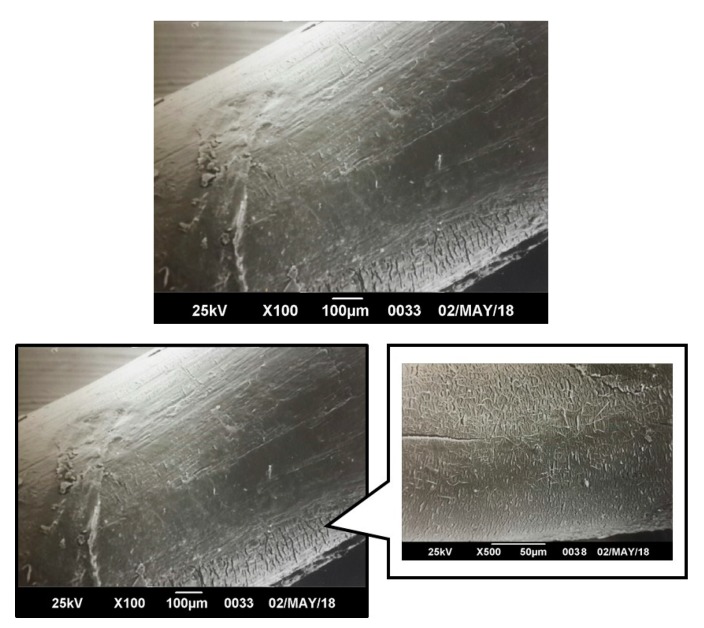
SEM micrographs of PLA 100 fiber surface morphology degraded for 12 weeks at pH = 8.50 and T = 37 °C, where a detail of surface degradation is shown.

Particularly for this process, it was seen that a circumferential propagation of the cracks happened, preferably, in a perpendicular direction to the axis of the fiber, which resulted in the formation of fragments with a completely smooth and flat cross section. Such behavior can be seen clearly seen in Figure 14 for PLA100 and slightly for PBAT100 fibers (T = 25 °C), and only for PLA100 fiber at the test temperature of 37 °C (Figure 15). Maybe released degradation products were dissolved in the reaction medium, resulting in a weight loss of the fiber due to the molecular mobility of the system caused by temperature [109,110]. In either case, the interstices or spaces initially occupied by these chain segments remained empty, and then the cracks were formed. These cracks started at the surface, and subsequently they propagated circumferentially to the interior of the fiber in a continuous manner, until they became fragmented. This results in a weight loss and in a decrease in the pH in the degradative medium, also decreasing the molecular weight, with a mechanical strength loss of the fibers. For the fibers degraded at 25 °C, only the PLA100 fiber showed evidence of the formation of these microcracks, being more pronounced and abundant for the testing temperature of 37 °C, and for an alkaline pH. This difference can be attributed to the fact that at a higher temperature, the kinetic of diffusion of the solvent was favored and accelerated the degradation process of the polymer. In the case of pH, it is widely known that PLA is a polymer that undergoes a hydrolysis attack preferentially in an alkaline medium [111].

Of the results shown above, PLA showed similar value of crystallinity than PBAT (Table 4). Crystals acts as barrier for gas and liquid diffusion. Hydrolysis takes place in the amorphous regions. However, the more linear structure of PLA makes this polymer more prone to hydrolysis. Compared to the fibers FC and F3, the fibers had higher values of Young´s modulus as indicative of higher stiffness of the fibers and, also, possible interactions between carbonyl groups mentioned in FTIR measurements makes surface erosion from the hydrolytic medium more difficult [106,112].

## 4. Conclusions

Blends made from PLA/PBAT improved the physical characteristics of the PLA. As the PBAT content in the blend increased, it became more flexible and elongation at break increased. From SEM micrographs of the blends and DSC results, a two-phase system was observed. The decrease in Young’s modulus was caused by addition of CEO, which acted as plasticizers for the polymer blend, evidenced in the SEM micrographs. The results for hydrolytic degradation showed a weight loss percentage for the fibers of below 5% during the 12-week process. The highest percentage was obtained for the fibers at 37 °C and alkaline medium (pH = 8.50). By week 12, a decrease in viscosity was observed for PLA100 and FC. From SEM micrographs, hydrolytic degradation was evidenced. The results suggest that the studied fibers subjected to hydrolytic degradation can be used in real conditions for a storage time of 12 weeks (3 months) and a temperature of 25 °C without changes in their morphology while keeping their structure and physical stability for applications in the conservation of shelf life of horticultural products in the form of nets including cinnamon essential oil as a antimicrobial agent. However, it will be useful to evaluate the mechanical properties of the fibers after hydrolytic degradation to assess the most appropriate application.

## Figures and Tables

**Table 1:** Composition of poly (lactic acid) (PLA) and poly (butylene adipate-co-terephthalate) (PBAT) in the blends.

Blend	PLA (wt %)	PBAT (wt %)
PLA100	100	0
PLA70	70	30
PLA60	60	40
PLA50	50	50
PBAT100	0	100

**Table 2:** Formulations for the preparation of the bio-based polymeric fibers, feeding speed for cinnamon essential oil (CEO), extruder, and take-off unit parameters.

Formulation	PLA60 (wt %)	CEO (wt %)	Feeding Speed CEO (g min^−1^)	Extruder (rpm)	Take-off Unit (rpm)
FC	100.0	0.0	0.00	20	32
F3	93.9	6.1	0.30	50	47
FG	95.7	0.0 (4.3 ^a^)	0.05	20	32

^a^ Glycerol.

**Table 3:** Degradation medium conditions.

Buffer	pH
Acetic acid	4.98
Phosphate	7.40
Ammonium	8.50

**Table 4:** FTIR characteristic absorption bands for CEO, PLA, and PBAT.

Compounds	Frequencies (Wavenumber in cm^−1^)	Assignment
CEO [44]	3267–2697	O-H stretching
	1672 and 1625	C=O stretching in aldehyde
	1574	C=C stretching skeletal vibration of the aromatic ring
	1452	C-OH bending
	1292	-CH_2_ swing in alkanes and =C-H in-plane bending of the aromatic ring
	1242	symmetric expansion of C-O-C of aromatic acid ester and vibrational stretching of C-OH from phenolics in eugenol from essential oils
	1122	C-O stretching and C-OH deformation
	971	C-H bending
	750	–CH bending out-of-plane in aromatic ring
	683	CH=CH bending out-of-plane in alkenes
PLA100 [45,46]	3034–2874	symmetrical and asymmetrical stretching of -CH in -CH_3_
	1745	C=O stretching
	1448	–CH in -CH_3_
	1178	O-C-O stretching
	1122, 1075, and 1050	C-O-C
	758	C-C- stretching
PBAT100 [47,48]	3003–2819	stretching of -CH in -CH_3_
	1709	C=O stretching
	1522–1336	C=O stretching for the phenolic group
	1187–982	C-O and C-O-C stretching vibrations
	730	–C-C- stretching

**Table 5:** Glass transition temperature, melting point, and crystallization temperature for the homopolymers, their blends, and the fibers.

Compound	T_g_ (°C)	T_c_ (°C)	T_m_ (°C)		Δ*H_m_* (J/g)	*X_c_* (%)
			PBAT	PLA		
PLA100	62.4	43.0	-	150.3	12.3	13.3
PLA70	61.8	65.0	123.7	149.2	11.1	8.3
PLA60	61.9	68.7	116.8	150.7	12.8	8.2
PLA50	61.4	75.1	126.0	149.5	13.2	7.1
PBAT100	−34.0 [42]	43.3	124.1	-	17.3	15.1
FC	61.2	74.8	130.5	149.6	12.2	7.9
F3	59.2	69.0	125.5	148.1	12.7	8.2

**Table 6:** Tensile properties of test specimens for homopolymers, their blends, and the fibers.

Compound	Young’s Modulus E (MPa) ^a^	Tensile Strength at Break σ (MPa) ^a^	Elongation at Break Ɛ (%)	Tenacity (kJ m^−3^) ^a^
PLA100	792.6 ± 29.8 e	52.4 ± 2.2 d	8.8 ± 0.2	72.3 ± 4.1 a
PLA70	534.8 ± 13.5 d	29.0 ± 2.0 c	14.8 ± 2.8	108.1 ± 1.8 a
PLA60	319.7 ± 32.6 c	13.5 ± 1.3 a	17.9 ± 1.9	45.2 ± 1.8 a
PLA50	232.2 ± 20.0 b	15.1 ± 1.2 ab	27.4 ± 1.2	101.4 ± 2.7 a
PBAT100	1.6 ± 0.2 a	17.7 ± 2.3 b	1207.6 ± 84.1	115,368.0 ± 6466.8 b
F3	2057.4 ± 62.5 b	32.9 ± 1.2 b	9.8 ± 0.1	261.0 ± 14.7 b
FC	2025.9 ± 23.4 b	34.6 ± 1.7 b	9.2 ± 0.8	193.4 ± 8.3 a
FG	1651.9 ± 73.3 a	26.6 ± 2.8 a	9.2 ± 1.1	173.3 ± 16.9 a

^a^ Different letter means statistically significant differences (*n* = 3, *p* ≤ 0.05).
